# Awareness and Perception of Healthcare Providers about Proxy Consent in Critical Care Research

**DOI:** 10.1155/2021/7614517

**Published:** 2021-09-30

**Authors:** Rania Mahafzah, Karem H. Alzoubi, Omar F. Khabour, Rana Abu-Farha

**Affiliations:** ^1^Department of Clinical Pharmacy, Jordan University of Science and Technology, Irbid 22110, Jordan; ^2^Department of Pharmacy Practice and Pharmacotherapeutics, University of Sharjah, Sharjah, UAE; ^3^Department of Medical Laboratory Sciences, Jordan University of Science and Technology, Irbid 22110, Jordan; ^4^Department of Clinical Pharmacy and Therapeutics, Faculty of Pharmacy, Applied Science Private University, Amman 11931, Jordan

## Abstract

**Objective:**

Proxy consent respects patients' autonomy when they are incapable of providing consent for research participation. Healthcare providers need to understand the ethical regulations and practices relevant to the proxy consent process. Thus, this study aimed to assess the knowledge and attitudes of healthcare providers about research proxy consent in the ICU setting.

**Methods:**

A cross-sectional survey-based design was used in the study. Study participants were resident and specialist physicians, registered nurses, and registered pharmacists from ICU units in Jordan. Participants were asked to fill out a questionnaire developed to assess their knowledge and attitudes towards informed proxy consent for research studies conducted at the ICU.

**Results:**

In this study, 145 healthcare providers completed the study questionnaire. The healthcare providers agreed that the purpose of the proxy consent is to inform the participants about the potential benefits (66.9%) and risks (66.9%) related to the research to study and respect patient's autonomy (44%), to discuss alternative options (62.1%), and to protect the researchers from any litigation (84.1%). Regarding the assessment of proxy consent, 65.5% of respondents believed that relatives are considered as an authorized legal representative for an informed consent decision on behalf of their ICU patients (65.5%) as they are knowledgeable about patients' values and preferences and have the desire to provide the necessary help. Respondents also agreed that the informed consent process should explain research protocols and procedures (76.6%), therapeutic alternatives (84.1%), potential benefits (41.4%), and potential risks (44.1%) and that participation in the research is voluntary (66.9%). No significant differences in the responses were found among different groups of healthcare providers.

**Conclusion:**

The majority of healthcare providers had inadequate awareness about the ethical aspects regarding the informed proxy consent process. Providing training regarding the informed consent process can improve the quality of the proxy consent process in clinical research studies in the ICU setting.

## 1. Introduction

Research in the intensive care unit (ICU) setting is essential to improve therapeutic options and the quality of provided services [1]. Informed consent from patients in ICU is one of the ethical research requirements [2, 3]. However, most ICU patients in Jordan frequently face life-threatening illnesses; they are usually unable to communicate due to sedatives, altered consciousness level, intubation, and mechanical ventilation [4].

Informed consent for clinical research studies is considered a significant challenge in critical care research. ICU patients usually cannot provide genuine informed consent for clinical research studies due to sedation or a change in the level of consciousness [4–7]. In such circumstances, proxy consent for clinical research studies is an acceptable method to protect those unable to provide consent for themselves [2, 8]. In Jordan, the human subject research studies are regulated and monitored by local Institutional Research Boards (IRBs), which consider ethical approval according to Helsinki's announcement for clinical trials, Guidelines for Good Clinical Practices (GCP), and Jordanian Clinical Research Law (JCRL) [9]. In empirical studies in the ICU setting, the Jordanian IRBs considered ethical approval to informed consent by relatives on behalf of the unconscious patients in the ICU [4, 10, 11].

Based on Jordanian Medical and Health Liability Law (2018), relatives are the authorized legal representatives for deciding on behalf of ICU patients [12]. Article 5 of JCRL states that the written consent should be obtained and signed from participants, but it does not mention the essential elements of the informed consent process. Article 8 of JCRL gives the responsibility for IRB to ensure the ethical considerations of research according to the Declaration of Helsinki and GCP [9, 13]. Based on the international ethical guideline, three fundamental aspects should be explained during the informed consent process: the voluntary nature of participation in research, the main benefit that will be achieved for future patients and society as a whole, and the potential incremental risks related to the research [2]; the researchers should fulfill these fundamental aspects to keep a high standard informed consent practices without deceived or coerced [2, 8].

The capacity to consent is a key principle in biomedical ethics to entitle the protection of vulnerable patients in the context of critical care research [14]. Unfortunately, some relatives intended to decline the consent because they are worried about their ICU patients taking part in clinical research studies [5]. On the contrary, other relatives consented to provide individual benefits for their ICU patients [15, 16]. Hence, informed consent from a person who does not have the mental capacity for decision-making is invalid [14]. Therefore, it is essential for healthcare professionals engaged in clinical research studies to be aware of the informed consent process and what information should be provided to the proxies to ensure human subjects' safety of human subjects [2, 17]. In the current study, we assessed the awareness of healthcare providers (physicians, nurses, and pharmacists) of fundamental informed consent among vulnerable ICU patients who have compromised ability to communicate or express their feelings, thoughts, and needs. Specifically, the study examined if the healthcare providers are aware of high standard practices of informed consent: [1] to respect the patient's autonomy and protect the individual from coercion and deception, [2] to reflect the best preferences and needs of the patients after understanding the core element of research [3], and to discuss the potential benefits and incremental risks from research.

## 2. Method

### 2.1. Study Design, Subjects, and Setting

Healthcare providers, including resident and specialist physicians, registered nurses, and registered pharmacists working at King Abdulla University Hospital (KAUH), were invited to participate in this cross-sectional survey-based study. King Abdulla University Hospital is one of the biggest teaching hospitals located in northern Jordan. This study was conducted over the period from March–June, 2019.

The researcher used G ^*∗*^ Power software version 3.1.9.7 to calculate the sample size. A 0.05 significance level, a power of 0.90, and a medium effect size of 0.30 required the minimum number of subjects to be 198. We have distributed 215 surveys. A total of 200 subjects filled the survey.

### 2.2. Questionnaire Construction

A questionnaire was developed to assess the knowledge and attitudes of healthcare providers towards informed proxy consent for research studies conducted at the ICU. The questionnaire was pilot tested in 20 participants to ensure quality and comprehensibility. Additionally, subjects from the pilot sample were asked to provide comments about how they understood each survey item to ensure content clarity and comprehension. Pilot samples were omitted from the final analysis. The reliability coefficient Chronbach alpha for all items of the study was >0.65. As for validity, the study survey was face validated via review by experts in the field, including a senior pharmacist, a physician, and an ethicist.

The survey consisted of four main sections (Supplementary Materials (available here)), the first section collects information regarding the demographics of the study participants. The second part is to assess participants' awareness about the purpose of informed consent for clinical research studies in critical care settings. The third section concerned participants' perception of obtaining proxy consent for clinical research studies from relatives of ICU patients. The last section evaluated participants' awareness about the information discussed during the proxy consent process. The last three sections were assessed using a three-level Likert scale (agree, neutral, and disagree), and each participant was allowed to choose only one choice as a response.

### 2.3. Data Collection

All participants were asked to complete the anonymous paper-based study questionnaire. Each participant was given a comprehensive description of the study aims and the definition of proxy consent according to local legal and national laws in Jordan without mentioning any details about the fundamental practices during the informed consent process. The survey was distributed by a trained researcher, who is also a clinical pharmacist. Participants were informed that the questionnaire could be completed within 5–10 minutes, and written informed consent was obtained from all study participants. Participants were given contact numbers of researchers and the human research ethics committee in case they decide to provide any concerns regarding the survey.

### 2.4. Ethical Consideration

The study protocol was approved by the Institutional Review Board (IRB) Committee at Jordan University of Science and Technology on 31^st^ January 2019 (reference no. 2/120/2019). The study was conducted following the ethical standards outlined in the World Medical Association Declaration of Helsinki guideline [2].

### 2.5. Statistical Analysis

Data were analyzed using Statistical Package for the Social Sciences (SPSS) version 21 (SPSS Inc., Chicago, IL, USA). The descriptive analysis was undertaken using the median and interquartile range for continuous variables and percentage for qualitative variables. Group differences for different categorical questions were tested using Pearson Chi-square test. *P* ≤ 0.05 was considered statistically significant.

## 3. Results

A total of 145 of the 200 approached healthcare providers completed the questionnaire giving a response rate of 72.5%. The sample consisted of 85 males (58.6%) and 60 females (41.4%). Physicians represent 36.6% of the study participants (*n* = 53), while 44.1% were nurses (*n* = 64) and 19.3% were pharmacists (*n* = 28). Around half of the participants were aged between 31 and 40 years (*n* = 70, 48.3%) and had more than 10 years of experience (*n* = 73, 50.3%). Details on the demographic characteristics of the study participants are presented in Table 1.

Table 2 reflected participants' awareness about the main purpose of obtaining informed consent in critical care research. The study respondents agreed that the purposes of the informed consent are to protect researchers from any medical litigation (*n* = 122, 84.1%), to inform patients about potential risks (*n* = 97, 66.9%) and benefits (*n* = 92, 63.4%) and to respect the patient's autonomy (*n* = 64, 44.1%). When comparing healthcare providers, physicians showed better awareness that informed consent is used to respect the patient's autonomy and to discuss the therapeutic options with the participants compared to pharmacists and nurses (*P* < 0.05).

The participants were asked about the reason to perform the informed consent from relatives (Table 3). 57.2% of respondents agreed that relatives are the best ones who could genuinely reflect the best interest of their patients' values and preferences. In comparison, 65.5% of the respondents (*n* = 95) agreed to informed consent by relatives only because they are the authorized legal representative based on Jordanian Medical Laws regardless of their capacity to make a decision. Moreover, only half of the respondents (*n* = 74, 51.0%) agreed that the relatives are the ones who desire to help their patients. No significant differences were found among the different groups of healthcare providers in their responses (*P* > 0.05).

Participants' awareness about the information that should be explained and discussed with proxies during the informed consent process was also assessed (Table 4). Results showed that 76.6% of them (*n* = 111) agreed that details about research protocols should be discussed in the consent, while less than half of the participants agreed that researchers provide information about incremental risks (*n* = 64, 44.1%) and benefits (*n* = 60, 41.4%) related to the research. Additionally, 84.1% of the respondents (*n* = 122) agreed that treatment alternatives should be discussed. Finally, 66.9% of the respondents (*n* = 97) agreed that consent should discuss the voluntary nature of the research and the ability of participants to withdraw from the research at any time. No significant differences were found among the different groups of healthcare providers in their responses (*P* > 0.05).

## 4. Discussion

The study is a pioneer and examined healthcare providers' awareness and perception towards informed proxy consent for clinical research studies in ICU settings in Jordan. Proxy consent provides a morally valid substituted judgment for participation in clinical research based on known patients' values and preferences [7]. Based on our results, healthcare providers were more likely to inform consent from relatives because they are the authorized legal representatives for deciding on ICU patients based on Jordanian Medical Law [16]. According to this, healthcare providers were more concerned about protecting themselves from medical litigation regardless of the capacity of relatives to make decisions.

Additionally, 76% of respondents agreed to discuss the research details; this does not follow the standard guideline [8]. Some proxies may not want to know extensive information, specifically in the critical care situation [18, 19]. A previous study demonstrated that most research participants were interested in learning only the major research complications, and it may be not possible for the researcher to know all the outcomes from research; thus, the researcher could discuss incremental nontherapeutic risks that may be happening compared with clinical practice, giving proxies the right to access more extensive information based on their discretion [20, 21].

Researchers should respect the high standard of the informed consent process to assess the validity of the proxy consent without being deceived or coerced [22]. In this study, healthcare providers were not fully filling the main purposes of informed consent. The healthcare providers should be comprehended more with ethical regulations and high standard proxy consent practices covered by the Belmont Report and the Declaration of Helsinki. The study alerts ethicists about potential ethical challenges related to the informed consent process. More training of healthcare providers on the informed consent process in clinical research can improve their understanding of the purposes of such a process.

This study showed that physicians are more aware of respecting participants' autonomy than other groups, and it may be due to the fact that physicians have more practical experience in developing protocols and obtained signatures for doing medical interventions; a comprehensive study is needed, probably using an unstructured survey to assess this. The main limitation of our study that must be pointed out is that it was conducted only in one institution, limiting the generalizability of the results obtained. Therefore, we strongly recommend further research be achieved using a larger sample size at multiple medical centers.

## 5. Conclusion

The findings of this study show that healthcare providers showed inadequate awareness about the legal and ethical aspects regarding the informed proxy consent process, which may affect the validity of the obtained consent. Thus, providing educational programs to healthcare providers is recommended to fill the gaps in their knowledge and awareness and improve the quality of this process.

## Figures and Tables

**Table 1:** Characteristics of the participated healthcare providers (*n* = 145).

Parameters	*n* (%)
Gender	
Male	85 (58.6)
Female	60 (41.4)
Age (years)	
20–30	29 (20.0)
31–40	70 (48.3)
≥41	46 (31.7)
Job title	
Physician	53 (36.6)
Pharmacist	28 (19.3)
Nurse	64 (44.1)
Years of experience	
1–5 years	24 (16.6)
6–10 years	48 (33.1)
More than 10 years	73 (50.3)

**Table 2:** Healthcare providers' awareness about the purposes of informed consent for clinical research in ICU setting (*n* = 145).

Purpose	Total (*n* = 145)	Physicians (*n* = 58)	Pharmacists (*n* = 23)	Nurses (*n* = 64)	*P* value^#^
	Percent agreed, *n* (%)				
To inform the participants about the potential risks related to the research study	97 (66.9)	35 (66.0)	19 (67.9)	43 (67.2)	0.791
To inform the participants about the potential benefits related to the research study	92 (63.4)	31 (58.5)	18 (64.3)	43(67.2)	0.630
To respect the patient's autonomy and protect the individual from coercion and deception	64 (44.1)	31 (58.5)	7 (25.0)	26 (40.6)	0.005^*∗*^
To discuss the alternative therapeutic options with the participants	90 (62.1)	43 (81.1)	10 (35.7)	37 (57.8)	<0.001^*∗*^
To protect the researchers from any medical litigation	122 (84.1)	46 (86.8)	23 (82.1)	53 (82.8)	0.944
To reduce the stress and anxiety related to participation in clinical research	56 (38.6)	23 (43.4)	6 (21.4)	27 (42.2)	0.791

#: using Pearson Chi-square test; ^*∗*^: significant at 0.05 significance level.

**Table 3:** Healthcare providers' perception towards obtaining the proxy consent for clinical research from relatives for ICU patients (*n* = 145).

Statements	Total (*n* = 145)	Physicians (*n* = 58)	Pharmacists (*n* = 23)	Nurses (*n* = 64)	*P* value^#^
	Percent agreed, *n* (%)				
Because the relatives of ICU patients are the ones who have knowledge about patients' values and preferences	83 (57.2)	34 (64.2)	15 (53.6)	34 (53.1)	0.457
Because the relatives of ICU patients have been recognized the authorized legal representative for informed consent decision on behalf of ICU patients	95 (65.5)	36 (67.9)	20 (71.4)	39 (60.9)	0.844
Because the relatives of ICU patients are the most one desired to help their patient through participating in clinical studies that may benefits their patients	74 (51.0)	23 (43.4)	14 (50.0)	37 (57.8)	0.625

#: using Pearson Chi-square test. ^*∗*^: significant at 0.05 significance level.

**Table 4:** Healthcare provider's awareness about the information that should be discussed during proxy consent process (*n* = 145).

Statements	Total (*n* = 145)	Physicians (*n* = 58)	Pharmacists (*n* = 23)	Nurses (*n* = 64)	*P* value^#^
	Percent agreed, *n* (%)				
Discuss all the details of research protocols and procedure	111 (76.6)	46 (86.8)	21 (75.0)	44 (68.8)	0.122
Discuss the potential benefits of the proposed research	60 (41.4)	21 (39.6)	13 (46.4)	26 (40.6)	0.908
Discuss other alternative therapeutic options	122 (84.1)	48 (90.6)	23 (82.1)	51 (79.7)	0.141
Discuss the incremental risks related to the research	64 (44.1)	23 (43.4)	11 (39.3)	30 (46.9)	0.044
States that participation in the research is voluntary and the participants can withdraw from the research at any time	97 (66.9)	36 (67.9)	18 (64.3)	43 (67.2)	0.924

#: using Pearson Chi-square test. ^*∗*^: significant at 0.05 significance level.

## Data Availability

All data generated or analyzed during this study are included in this published article.

## Supplementary Materials

Supplementary Materials S1: the study survey.
