# Supplementary material for: Awareness and Perception of Healthcare Providers about Proxy Consent in Critical Care Research
Source: Crit Care Res Pract. 2021 Sep 30;2021:7614517. doi: 10.1155/2021/7614517 (PMC8497167; doi:10.1155/2021/7614517)
Supplement: Supplementary Materials — S1: the study survey. [file 7614517.f1.docx]

**Questionnaire**

**Study Title**: Awareness and Perception of Healthcare Providers about Proxy Consent in Critical Care Research

**An introduction**:

You are invited to participate in a research study onto evaluate healthcare provider's perceptions about obtaining proxy consent from the patient's authorized family members rather than the patient in the intensive care unit to participate in clinical research.

Before agreeing to participate in this study, it is important to read and understand this form.

**Study purpose:**

This study aims to evaluate the perceptions of healthcare providers about taking the consent from the patient's relatives who are authorized to make the decision instead of the patient in the intensive care unit to participate in a clinical research.

**Study Procedures:**

If you agree to participate in this study, you will be asked to fill-out a questionnaire that takes only a few minutes. This questionnaire is divided into two parts: the first part will ask you to provide demographic information and the second part is prepared to assess the healthcare provider's perceptions about obtaining consent from the patient's family to participate in the research.

**Participation in the search:**

Your participation in this study is voluntary and you have the full right to refuse to participate in this study without any consequences.

**Benefits that may result from this study:**

It is expected that this study will benefit decision makers in scientific research committees, scientific research ethics, medical researchers and healthcare providers in Jordan in the future.

**Privacy and Confidentiality:**

You are not required to give out any private information. Also, the data from this study will be used for scientific research purposes and nothing will identify you individually.

| **Part 1:**Demographic characteristics of health-care providers |
| --- |

Gender

- Male
- Female

Age (years)

- 20-30
- 31-40
- ≥ 41

Job title

- Physician
- Pharmacist
- Nurse

Years of Experience

- 1-5 years
- 6-10 years
- More than 10 years

| **Part 2:** Health-care providers perceptions about proxy consent |
| --- |

| **First domain:** Health-care providers’ awareness about the purposes of informed consent for clinical research in ICU setting | | | | | |
| --- | --- | --- | --- | --- | --- |
| **Strongly agree** | **Agree** | **Neutral** | **Disagree** | **Strongly disagree** | **Statement** |
|  |  |  |  |  | To inform the participants about the potential risks related to the research study. |
|  |  |  |  |  | To inform the participants about the potential benefits related to the research study. |
|  |  |  |  |  | To respect the patient's autonomy and protect the individual from coercion and deception. |
|  |  |  |  |  | To discuss the alternative therapeutic options with the participants |
|  |  |  |  |  | To protect the researchers from any medical litigation |
|  |  |  |  |  | To reduce the stress and anxiety related to participation in clinical research. |

| **Second domain:** Health-care providers’ perception towards obtaining the proxy consent for clinical research from relatives for ICU patients | | | | | |
| --- | --- | --- | --- | --- | --- |
| **Strongly agree** | **Agree** | **Neutral** | **Disagree** | **Strongly disagree** | **Statement** |
|  |  |  |  |  | Because the relatives of ICU patients are the ones who have knowledge about patients' values and preferences. |
|  |  |  |  |  | Because the relatives of ICU patients are recognized the authorized legal representative for informed consent decision on behalf of ICU patients. |
|  |  |  |  |  | Because the relatives of ICU patients are the most one desire to help their patient through participating in clinical studies that may benefits their patients. |

| **Third domain:** Health-care provider’s awareness about the information that should be discussed during proxy consent process | | | | | |
| --- | --- | --- | --- | --- | --- |
| **Strongly agree** | **Agree** | **Neutral** | **Disagree** | **Strongly disagree** | **Statement** |
|  |  |  |  |  | Discuss all the details of research protocols and procedure. |
|  |  |  |  |  | Discuss the potential benefits of proposed research. |
|  |  |  |  |  | Discuss other alternative therapeutic options. |
|  |  |  |  |  | Discuss the incremental risks related to the research. |
|  |  |  |  |  | States that participation in the research is voluntary and the participants can withdraw from the research at any time. |
